# Caregiver Reports of Patient-Initiated Violence in Psychosis

**DOI:** 10.1177/070674371405900705

**Published:** 2014-07

**Authors:** Juliana Onwumere, Sarah Grice, Philippa Garety, Paul Bebbington, Graham Dunn, Daniel Freeman, David Fowler, Elizabeth Kuipers

**Affiliations:** 1 Lecturer in Clinical Psychology, Institute of Psychiatry, King’s College London, London, England.; 2 Clinical Psychologist, Institute of Psychiatry, King’s College London, London, England.; 3 Professor of Clinical Psychology, Institute of Psychiatry, King’s College London, London, England; Professor of Clinical Psychology, Biomedical Research Centre, South London and Maudsley National Health Service Foundation Trust, Institute of Psychiatry, King’s College London, London, England.; 4 Emeritus Professor of Social and Community Psychiatry, Mental Health Science Unit, University College London, London, England.; 5 Professor of Biomedical Statistics, Centre for Biostatistics, University of Manchester, Manchester, England.; 6 Professor of Clinical Psychology, Department of Psychiatry, University of Oxford, Oxford, England.; 7 Professor of Social Psychiatry, Department of Psychiatry, University of East Anglia, Norfolk, England.

**Keywords:** caregivers, psychosis, violence

## Abstract

**Objective:**

Aggressive behaviour in psychosis is not uncommon. Community provision for people with psychosis has left informal caregivers to take on a greater role in their care. However, few studies have explored links between patient-initiated violence in mental health caregiving relationships and caregiver functioning. Our study investigated caregiver reports of aggressive acts committed by their relative with psychosis and their links to caregiver appraisals of the caregiving relationship and caregiver outcomes.

**Method:**

Caregivers of patients with a recent relapse of psychosis, recruited to a psychological therapy trial, completed the audiotaped Camberwell Family Interview at baseline. This semi-structured interview includes questions on the quality of the relationship between caregiver and patient, and patient history of violence. Seventy-two transcripts of interviews were assessed for reports of patient-initiated violence.

**Results:**

One-half of the caregiver sample (52.9%) reported an incident of patient-initiated violence during their interview; 62.2% of these involved violence toward themselves, and 24.3% toward property. Reports of patient violence were associated with caregiver ratings of hostility expressed toward patients, lower self-esteem, and emotion-focused coping. People caring on their own were more likely to report incidents of patient violence. Younger patients, males, and inpatients were more frequently identified as having a history of this kind of violence.

**Conclusions:**

Our findings suggested that caregiver reports of patient-initiated violence in psychosis are not uncommon. Mental health staff need to be aware of the risks of such violence for caregivers of people with psychosis, and consider appropriate procedures for minimizing it.

**Clinical Trial Registration Number:** ISRCTN83557988

## Abstract (fr)

**Objectif:**

Le comportement agressif n’est pas rare dans la psychose. Les services communautaires pour les personnes souffrant de psychose ont laissé des soignants naturels assumer un rôle plus important dans leurs soins. Toutefois, peu d’études ont exploré les liens entre la violence initiée par les patients dans les relations de soins de santé mentale et le fonctionnement des soignants. Notre étude a examiné les rapports de soignants sur des actes agressifs commis par leur parent souffrant de psychose et leurs liens avec les évaluations des soignants de la relation de soins et des résultats pour les soignants.

**Méthode:**

Les soignants de patients ayant eu une rechute de psychose récente, recrutés pour un essai de thérapie psychologique, ont répondu à l’Entrevue de famille de Camberwell sur bande sonore, au départ. Cette entrevue semi-structurée comprend des questions sur la qualité de la relation entre le soignant et le patient, et sur les antécédents de violence du patient. Soixante-douze transcriptions des entrevues ont été évaluées pour des rapports de violence initiée par le patient.

**Résultats:**

La moitié de l’échantillon des soignants (52,9 %) a rapporté un incident de violence initiée par le patient durant leur entrevue; 62,2 % de ces incidents impliquaient la violence envers eux-mêmes, et 24,3 %, envers des biens. Les rapports sur la violence des patients étaient associés à des cotes d’hostilité des soignants exprimée à l’endroit des patients, à une faible estime de soi, et à l’adaptation axée sur les émotions. Les personnes soignant de leur propre initiative étaient plus susceptibles de rapporter des incidents de violence des patients. Les patients jeunes, les hommes, et les patients hospitalisés étaient plus fréquemment identifiés comme ayant des antécédents de ce type de violence.

**Conclusions:**

Nos résultats suggèrent que les rapports de soignants sur la violence initiée par les patients dans la psychose ne sont pas rares. Le personnel de la santé mentale doit être conscient des risques de cette violence pour les soignants de personnes souffrant de psychose, et envisager des procédures appropriées pour la réduire.

A global program of deinstitutionalization and gradual transfer of inpatient care to community settings has meant that caregivers are taking on increasing levels of caregiving duties and are widely recognized for their input to patient care and recovery.^1^ People with psychosis who have caregivers, compared with those without, tend to report more favourable outcomes, including improved treatment engagement, fewer inpatient admissions, and increased quality of life.^2^–^5^

While many caregivers can report positive experiences as part of their role,^6^,^7^ their physical and mental health can be negatively affected.^8^–^10^ Perlick et al^10^ found that almost two-thirds of the 264 mental health caregivers they assessed suffered from at least 1 medical condition, such as hypertension and arthritis. Caregivers also commonly experience a broad range of negative emotional states, including loss, guilt, anger, fear, shame, and anxiety. They can report high levels of caregiver burden,^8^,^11^ restricted social networks with low levels of social support,^12^ and trauma symptoms.^13^ About 30% to 40% of caregivers report clinical levels of depression.^14^,^15^ Emotional and physical burnout recorded in caregivers has been equivalent to levels observed in psychiatric staff.^16^ Caregiver distress is associated with less adaptive coping styles characterized by behavioural and cognitive avoidance.^17^,^18^

Caregiving relationships in psychosis have traditionally been assessed using the EE index.^19^ EE is a measure of the family environment and described as a caregiver’s evaluation of the quality of their relationship with the patient.^20^ Caregivers defined as having high EE report above threshold levels of criticism and (or) hostility and (or) overinvolvement toward patients. High EE is positively related to avoidant coping, negative caregiving experiences (burden), and poorer levels of social support, independent of patient symptomatology.^9^ EE remains of interest to clinicians because high levels serve as robust predictors of poorer patient outcomes, including increased rates of patient relapse and inpatient readmissions.^21^–^23^

**Clinical Implications**

- Carergivers may benefit from interventions that promote their safety through developing skills in violence resolution, problem-solving, and identifying and managing early warning signs of patient violence.
- Patients may benefit from CBT interventions to address anger management issues.
- Assessing caregivers’ experience of patient-initiated violence as part of routine risk assessment, should be considered.

**Limitations**

- Accuracy checks on caregiver reports of patient violence were not undertaken.
- Our study employed a cross-sectional design, which precludes causal inferences being made.
- Caregivers were predominately white, middle-aged females who were the mothers of the patients who had recently relapsed and were recruited as part of a psychological therapies trial. Caution must be taken in generalizing current findings to other caregiver groups.

In comparison of caregivers’ experience of violent acts committed by their relative with psychosis, with the general population, people with psychosis are statistically more likely to be victims of violence.^24^–^27^ However, they also perpetrate acts of violence.^28^,^29^ This is particularly evident during the FEP^30^–^32^ and during the first year of illness.^33^ Data from a large-scale United Kingdom study of FEP cases indicated that nearly 40% of participants were aggressive at first service contact, with more than one-half reported as being physically violent.^32^ Similarly, in a small study of service users attending a routine service for people with at-risk psychosis mental states, 38% had a history of violent behaviour. Methodological limitations inherent in many studies exploring violence and psychosis (for example, definitional issues or lack of adequate control group) can influence the conclusions drawn about associations and risk factors.^34^ However, several risk factors for violence in patients with psychosis have been identified; these include younger age, polysubstance use, ethnicity, mania symptoms, patient appraisals of personal threat, and the experience of thoughts that override their sense of control.^32^,^35^,^36^ Caregivers, particularly mothers who live with patients, are more likely than members of the general public to be the target of violent acts committed by their relative with psychosis^30^,^37^–^39^ and withstand more severe injuries.^39^

Loughland et al^40^ reported that during the preceding 12 months, almost 80% of caregivers sampled had experienced moderate to severe levels of aggression from their relatives with psychosis, which was mainly in the form of verbal aggression. More than one-fifth also reported having feared for their lives and expressed concern about the violence reoccurring. Chan^41^ found that over one-third (37.7%) of caregivers of people with a diagnosis of schizophrenia reported at least one incident of severe physical assault and one-half (52.4%) reported a minor physical assault. In a study^42^ of service users with psychosis in a community mental health team, at least 40% of caregivers had been threatened with violence from the service user since illness onset; among whom, 40% had been struck, 17% had sustained a physical injury, and 40% reported that the patient had destroyed property. In addition, 66% of caregivers reported that the patient regularly lost their temper. Reports of verbal and physical abuse in patients were positively correlated.^42^ Data taken from a small qualitative investigation suggested that caregivers may minimize or fail to appreciate the degree of threat posed by the patient’s aggression.^43^ Despite this literature, the links between patient-initiated violence and caregiver outcomes in psychosis have rarely been investigated. A small body of evidence suggests that verbal and physical aggression from patients are positively related to trauma symptoms in caregivers.^40^,^44^ Reported physical abuse by patients also shares a positive relation with caregiver reports of burden, distress, and patient-focused criticism.^45^ There have been no investigations of how patient-initiated violence relates to caregiver attributes (for example, coping styles) that are already known to impact on caregiver and patient outcomes.^9^,^17^,^23^

Our study aimed to use the semi-structured CFI,^46^ to investigate caregivers’ reports of patient-initiated violence and their links with caregiver characteristics. We predicted that reports of violence would be associated with more negative carer–patient relationships (high EE), poorer well-being (more burden and distress), dysfunctional (avoidant) coping styles, and lower self-esteem.

## Method

### Participants

Participants and their caregivers were recruited to the PRP Trial.^5^ This was a multi-centre British randomized controlled trial of CBT and family intervention for psychosis. All participants provided written informed consent and the South Thames Multi-Centre Research Ethics Committee provided ethical approval of the study. Details of the trial participants, method and results have been published elsewhere.^5^ Patients were recruited at the time of relapse in their positive symptoms. Patients in contact with caregivers for at least 10 hours a week were asked for consent to approach them. Caregivers were identified as the parents, spouses, or partners living with the patient, other acknowledged caregivers living with the patient, or acknowledged caregivers not living with the patient but in contact at least 3 times and 10 hours a week. Caregivers had to speak English adequately to complete the assessments.

One hundred twenty-five patients who were eligible to participate did not consent. No data exist on the suitability of their caregivers, though comparisons between patients who consented and those who did not showed that consenting patients were more likely to be men (χ^2^ = 8.23, *df* = 1, *P* = 0.004), with a history of voluntary admissions (χ^2^ = 17.2, *df* = 1, *P* < 0.01). Fewer had a history of violence (χ^2^ = 11.3, *df* = 1, *P* = 0.001) or sexual offences (χ2 = 7.43, *df* = 1, *P* = 0.006). Ninety-four patients with eligible caregivers consented to enter the trial. Eleven caregivers did not consent, although 3 consented for their initial assessments. Our study was comprised of 72 caregivers who provided complete CFI audiotapes that were fully transcribed.

### Caregivers Measures

#### Camberwell Family Interview

This semi-structured audiotaped interview^46^ involves asking caregivers about their relationship with the patient, including a specific question about whether the patient had ever been violent in the past. EE ratings consist of critical comments (frequency count), hostility (0 to 3), emotional overinvolvement (0 to 5), positive remarks (frequency count), and warmth (0 to 5). Caregivers are defined as high EE if they make 6 or more critical comments, and (or) score 1 or more on hostility, and (or) score 3 or more on emotional overinvolvement. EE ratings were also examined as continuous variables. Ratings were made at the time of the initial investigation by trained assessors blind to the predictions of our study.

#### Ratings of Patient-Initiated Violence From the Camberwell Family Interview

Ratings of caregivers reports of patient-initiated violence were made by 2 independent raters from transcripts of CFI audiotapes. Violence was defined as any aggressive act toward another person or property. Verbal aggression was not included. The Kappa statistic for violence ratings indicated excellent agreement (0.91). Raters were blind to EE ratings, and to previous ratings of carer mood, burden, and support.

#### The General Health Questionnaire

The GHQ-28^47^ was used as a measure of caregiver stress and is a scaled version of the GHQ. In our study, we used the GHQ-28 total scores, and the scoring was based on the 0, 1, 2, 3 method. Higher scores indicate greater levels of distress.

#### The Experience of Caregiving Inventory

The ECI^48^ is a 66-item, self-report questionnaire developed to assess the subjective negative and positive experiences of caregiving. Respondents rate how often they have thought about a particular issue in the last month prior to completing the questionnaire on a 5-point scale (range: 0 never to 4 nearly always). Negative caregiving appraisal is calculated from the sum of the 8 negative ECI subscales, and positive appraisal from the sum of the 2 positive ECI subscales. The scale has good reliability and validity.

#### Abbreviated Coping Orientations to Problems Experienced Inventory

The COPE Inventory^49^,^50^ assesses an extensive range of functional and dysfunctional coping styles on a 4-point Likert scale of frequency of use. It comprises 15 distinct scales; the total scores for each scale are calculated by summing individual items. The abbreviated COPE includes all the scales but has 2 questions per scale instead of the usual 4. Our study used the composite avoidant coping scale, which comprises the behavioural and mental disengagement, alcohol or drug use, and denial subscales, and the support seeking and active coping scales. The COPE has proven reliability and validity.^49^,^50^

#### Confidante Question

An indication of social support and network was assessed by having carergivers answer yes or no to the following question: “Do you have someone in whom you can confide?” Similar questions have been used with other psychosis caregiving studies^51^ and in physical health.^52^

#### Rosenberg Self-Esteem Scale

This self-report measure^53^ assesses current levels of global self-esteem. It comprises 10 items measured on a 4-point Likert scale (strongly agree to strongly disagree). Higher scores are indicative of low global self-esteem.

#### Patient Measures

##### The Positive and Negative Syndrome Scale

The PANSS^54^ is a 30-item, semi-structured interview designed to rate psychotic symptomatology in relation to the last 72 hours. It has 3 subscales: positive symptoms, negative symptoms, and general psychopathology. All items are rated on a 7-point Likert scale representing increasing levels of psychopathology. Higher scores indicate higher levels of symptomatology.

##### Beck Depression Inventory-II

The BDI-II^55^ is a well-established, 21-item, 4-point, self-report measure for the assessment of depression in the previous 2 weeks.

##### Beck Anxiety Inventory

The BAI^56^ is a self-report, 21-item, 4-point measure used for the assessment of common anxiety symptoms for the past week.

### Statistical Analysis

Data were analysed with SPSS for Windows, version 17.0 (SSPS Inc, Chicago, IL).^57^ Statistical tests were 2-tailed, with an alpha level of 0.05.

## Results

Table 1 summarizes the caregiver and patient socio-demographic data and Table 2 presents a summary of the caregiver clinical characteristics.

Caregiver participants were mainly female, aged in their early 50s, and at least one-third were unemployed. Almost two-thirds were in partnerships. Just under 70% were co-resident with the patient and were related through being their parent or partner. Patients were mainly inpatients. They were also male, single, and at 80%, most were unemployed. Patients had an average 11.5 year illness history.

### Caregivers Reports of Patient-Initiated Violence

About one-half of the caregivers reported at least 1 incident of patient-initiated violence during their CFI interview (52.9%, *n* = 38). Among these, 62.2% reported personal violence, 5.4% of the violence was toward other family members, 8.1% was toward people outside the family, and 24.3% was toward property.

Patient violence included incidents causing injury requiring hospital treatment: assaults involving the use of a weapon and property destruction including acts of arson. Examples are listed in Table 3.

### Caregivers Correlates of Patient-Initiated Violence

This was predominantly a low EE carergiver sample (67%). While overall EE status was not related to violence (Fisher exact test *P* = 0.447), the hostility component was greater in carergivers reporting violence (*t* = 2.201, *df* = 68, *P* = 0.03, mean difference = –0.34 [95% CI −0.663 to −0.032]).

Patient violence was unrelated to carergiver burden or carergiver distress (*P* > 0.05). Self-esteem was significantly lower in carergivers reporting patient violence (*t* = 2.199, *df* = 60, *P* = 0.03, mean difference = −3.10 [95% CI −5.92 to −0.281]), while levels of functional coping through emotional support were significantly higher (*t* = 2.902, *df* = 61, *P* = 0.005, mean difference = −0.56 [95% CI = −0.956 to −0.176]). No other differences in coping styles were found (*P* > 0.05). Reports of patient violence were unrelated to carergiver sex, age, and employment status, and whether carers and patients lived together (Fisher exact test *P* > 0.05). However, patient violence was more likely to be reported by a person on their own caring for a patient (Fisher exact test, *P* = 0.03).

### Patient Correlates of Violence

Violence was significantly higher in patients who were younger (*t* = 2.418, *df* = 68, *P* = 0.02, mean difference = 6.71 [95% CI 1.174 to 12.263]); male (Fisher exact test *P* = 0.04); single (Fisher exact test *P* = 0.047); and those who were inpatients at the time of recruitment into the study (Fisher exact test *P* = 0.02).

## Discussion

Our study investigated caregiver reports of patient-initiated violence in psychosis using transcripts from the CFI.^46^ The carergiver sample was predominately comprised of white, middle-aged females. Consistent with other reports,^40^,^41^ just over one-half of the carergiver sample reported incidents of patient-initiated violence, of which nearly two-thirds were directed toward the carergiver. Patient-initiated violence was associated with higher levels of carergivers’ expressed hostility toward patients (a component of high EE), their lower self-esteem, and greater reported use of emotional support styles of coping. Patient violence was more likely to be reported by a person on their own caring for the patient. The results offered mixed support for our hypotheses.

EE hostility reflects a carergiver’s extreme criticism of the patient’s personality and character. Our findings are consistent with previous work linking carergiver criticism and patient-initiated violence.^41^ It is perhaps unsurprising that carergivers perceive patients with a history of violence more negatively. Given that most violence was directed at carergivers, links with low self-esteem in carergivers are also plausible. We know that patient violence has a detrimental impact on the well-being and functioning of formal carergivers (that is, paid mental health staff), which can include sleep disturbances, stress, and posttraumatic stress disorder.^58^ It is also linked to burnout and low morale in staff.^59^ Poor self-esteem has also been associated with reports of intimate partner violence.^60^ The negative relation between patient violence and low carergiver self-esteem in our study are in line with such findings.

Contrary to our predictions and previous studies,^42^ but consistent with Loughland et al,^40^ we found no relation between carergiver distress, burden, and reports of patient violence. However, carergivers exposed to violence were more likely to seek emotional support from trusted others. This may help to manage possible feelings of shame and embarrassment, and buffer distress. The literature on domestic violence offers some support for this explanation.^60^ Interestingly, carergivers reporting violence were not more likely to engage in avoidant (dysfunctional) coping strategies.

Our results indicated that some carergivers may be at greater risk of patient-initiated violence than others. For example, carergivers defined as being on their own, being single (used here as a measure of isolation), reported more patient violence. It could be argued that carergivers on their own have fewer opportunities to access immediate support in their day-to-day caregiving role, and the presence of another adult may discourage violent incidents from occurring or escalating. Caregiving relationships have the propensity to be more intense when the caregiver is on their own, and it may be easier for carergivers to become the targets of patient’s distress or unusual beliefs when they are on their own, or in more contact with the patient.^40^

## Clinical Implications

The informal caregiving role in psychosis is often challenging, with few opportunities afforded for respite. Carergivers often take on their role with no preparation time, and little training, guidance, or specialist knowledge about the illness and its impact on the patient. They can often become isolated by feelings of stigma and shame.

Carergivers are likely to benefit from interventions that promote their safety, by developing skills in violence resolution and problem-solving, and in identifying and managing warning signs, such as acute affective or psychotic symptoms, and promoting adaptive coping strategies that explicitly include pathways of how and where to access support and reduce or prevent risk.^61^ Equally, patients may benefit from CBT interventions to address anger management issues, early warning signs, and (or) the negative emotional sequelae that may follow their behaviour, such as regret, shame, guilt, and stigma.^62^

In line with recommendations reported for domestic violence and mental health,^63^ the findings from our study indicate the importance of general inquiry about carergivers’ experience of patient-initiated violence as part of routine risk assessment. The training implications for mental health staff must also be considered.^64^,^65^

## Limitations

First, our study used indirect information from responses obtained during previously completed CFIs, and answers were not necessarily followed up, and accuracy checks on carer reports were not available. Therefore, some reports lack detail (for example, frequency of behaviours), and it is possible that our figures underestimate the actual level of violence. Exploration of the differences between carergivers who had been victims of patient-initiated violence to those who had not would have also extended our understanding of the potential impact. The patient perspective was not sought, and carergivers were not asked about carer-initiated violence, which can be an issue in other caregiving relationships.^66^ However, given the typical caregiving relationship in psychosis (that is, middle-aged mother caring for adult son) the risk of carergiver violence might be assumed to be comparatively less. Second, the cross-sectional design limits causal inference. However, the findings are in line with the available literature on intra-familial violence by patients with psychosis.^41^ Nevertheless, it is possible that carergiver hostility and low self-esteem precede incidents of patient violence. A prospective study would help to establish the causal direction of these links. Likewise, efforts to determine the pattern of relations between carergiver self-esteem and reports of patient violence and victimization are indicated as are investigations of the links between carergiver functioning and the type and severity of patient-initiated violence experienced. Third, while the sample is consistent with those described in other caregiver studies,^40^,^67^ the carergivers were predominately white, middle-aged females who were the mothers of the identified service user who had recently relapsed and recruited as part of a psychological therapies trial. Caution is therefore required in generalizing the findings to other carergiver subgroups (for example, carergivers drawn from black and minority ethnic groups, or spousal or sibling carergivers). Finally, the lack of a nonclinical control group precludes discussion about how common the experience of patient-initiated violence is and what, if any, are the specific issues related to a psychosis caregiving group.

## Conclusion

Patient violence may impact on carergiver self-image and the caregiving relationship. Given the difficulty of disclosure of violence in close relationships and the lack of direct questioning in our study, mental health staff need to be able to explore effective ways to assess such issues and intervene appropriately.

## Figures and Tables

**Table 1:** Caregiver and patient social, demographic, and clinical characteristics

Demographic	Patient	Caregivers
Sex, female, %	27.1	72
Age, years, mean (SD)	35.8 (12.0)	52.9 (12.9)
Length of illness, years, mean (SD)	11.5 (9.6)	
Inpatient or outpatient, %	58.6 or 41.4	
Marital status, %		
Single	65.2	11.8
Married	24.6	60.3
Divorced or separated	8.7	13.2
Widowed	1.4	10.0
Cohabiting		2.9
Other		1.5
Relationship to patient, %		
Parent		55.1
Partner		34.8
Sibling		8.7
Child		1.4
Living with patient, yes, %		69.8
Hours of weekly face-to-face patient contact, mean (SD)		38.5 (22.7)
Ethnicity, %		
White	85.6	89.7
Black	5.9	4.4
Other	8.5	5.9
Employment status, %		
Unemployed	80.0	34.8
Employed	12.9	40.9
Other	7.2	24.3

**Table 2:** Clinical characteristics of the caregiver sample

Caregiver variable	Mean (SD)
Criticism	3.27 (3.51)
Hostility	0.21 (0.67)
EOI	1.71 (1.13)
Warmth	2.22 (1.16)
Positive comments	1.95 (1.97)
Low EE or high EE, %	67.1 or 32.9
Distress, GHQ	25.2 (13.7)
ECI burden	92.8 (29.9)
ECI positive	30.8 (7.78)
Self-esteem, RSES	19.2 (5.70)
Avoidant	14.7 (3.9)
Active coping	2.72 (0.73)
Emotional support	2.67 (0.81)
Instrumental support	2.38 (0.76)
Confidante, yes or no, %	84.4

ECI = Experience of Caregiving Inventory;

EE = expressed emotion; EOI = emotional over involvement;

GHQ = General Health Questionnaire;

RSES = Rosenberg Self-Esteem Scale

**Table 3:** Examples of caregiver reports of patient violence from the Camberwell Family Interview

Violence toward caregiver	Violence toward others	Violence toward property
He used to give you the odd punch and kick as he was walking by, but his mind was so confused and muddled. She did hit me a few times when we used to live together. He started to slap me, threaten me, and push me. This went on for a couple of days and I couldn’t get anyone out to assess him. I have passed him money in the past he squeezed it in my hand that made it bleed. He twisted my hands as if I have done something wrong and that does really get to me. He only ever lashed out at me at once. She hit me yesterday because she wanted me to come up here every day like I used to. He punched me in the face and threatened to kill me. I had a fractured rib. There was a period when he was violent every day. The day she hit me I was going to ring child line. This wasn’t my mum. This is because she had escaped from hospital. Only been aggressive to me once, years ago. I said if you do that again I will hit you in the balls when you are fast asleep… he has never touched me since. He has got physical with my eldest daughter—he transferred it over to her.	He was really violent and he ended up having to go to court for actual bodily harm and he got 240 hours community service. The only reason he didn’t go to prison was because he was actually admitted to hospital and he got the help he needed because he had stopped taking his medication. He had attacked his sister and we have heard about him attacking other people. He started beating up my wife and being abusive to her and it just got out of hand. Only after he put a man in hospital that we knew he was hearing voices—the man pushed past him and the voices told him to kill him. He had attacked somebody in the street with the knife and he was also imagining himself as God. Came back from work and found her unconscious, and it was actually clear that he had tried to kill her.	She smashed things—she had pictures she smashed. One night he woke up and punched the wall right next to my head. I thought I would just go downstairs and sleep on the settee. He would smash a chair. He smashed up a shop. He’d smash glass. He took the car and smashed the car. He got a paving slab and slung it at the car—he was taking it out on the car—that was one of the first signs. Anything he felt voices were coming from, he has smashed up, televisions and telephones. He does lose his temper but not as bad as he used to be—he would punch holes in the doors and smash windows in. Burned down his bed. The next morning he jumped out of bed, went into the bathroom, and smashed the mirror. Set fire to my living room, but you cannot say nothing to him.

## Abbreviations

BAI: Beck Anxiety Inventory
BDI: Beck Depression Inventory
CBT: cognitive-behavioural therapy
COPE: Coping Orientations to Problems Experienced
CFI: Camberwell Family Interview
ECI: Experience of Caregiving Inventory
EE: expressed emotion
FEP: first-episode psychosis
GHQ: General Health Questionnaire
PANSS: Positive and Negative Syndrome Scale
PRP: Psychological Prevention of Relapse in Psychosis
